# Intensive Insulin Therapy in Intensive Care: An Example of the Struggle to Implement Evidence-Based Medicine

**DOI:** 10.1371/journal.pmed.0030456

**Published:** 2006-12-05

**Authors:** Marcus J Schultz, Annick A. N. M Royakkers, Marcel Levi, Hazra S Moeniralam, Peter E Spronk

## Abstract

Schultz and colleagues discuss the factors hindering implementation of intensive insulin therapy.

A long with improving patients' safety and reducing medical errors, one of the main challenges in medicine is implementing new strategies that have the potential to improve health outcomes. After the process of critically appraising clinical trials has finished, and the results of this appraisal are used to guide changes in clinical practice, it is then time to critically appraise the success of implementation.

In other words, are physicians really performing the new strategy in its entirety? If they are not, what are the barriers to implementation? Unfortunately, there is no “golden bullet” for successful implementation of new strategies in medicine [1,2]. However, common factors in the failure of implementation have been identified, including environmental factors and factors related to the strategy itself [3].

Critically ill patients without diabetes often develop hyperglycemia. Until recently, it was common practice to treat only marked hyperglycemia in these patients, since hyperglycemia was considered to be an adaptive response to critical illness. But clinical trials have shown that so-called intensive insulin therapy (IIT) aiming at normoglycemia (i.e., blood glucose concentrations [BGC] between 80–110 mg/dl) can significantly decrease mortality and morbidity of patients in the surgical and medical intensive care unit (ICU) [4–7].

We questioned whether IIT truly has become part of standard therapy in ICU patients and, if it is applied, to what extent? We performed a systematic search of the medical literature, in which we focused on surveys and reports on the practice of ITT (see Text S1). We searched for reasons why IIT had not been implemented. We compared factors that hindered implementation of IIT with factors hindering the adoption of other recently introduced strategies, both in ICU medicine and general medicine.

## Current Recommendations on IIT and Feared Complications

Following publication of the first randomized controlled trial of IIT by van den Berghe and colleagues [4], several groups have recommended IIT as the standard of care for those who are critically ill. These groups include the Joint Commission on Accreditation of Healthcare Organization (http://www.jcaho.org), the Institute for Healthcare Improvement (http://www.ihi.org), and the Volunteer Hospital Organization (http://www.vha.com). In addition, IIT is promoted as a part of a care bundle for sepsis by the American Thoracic Society (http://www.thoracic.org) and experts in the field [8]. Also, IIT has become, to some extent, a benchmark for the quality of ICU care [9].

However, over the last few years, a number of commentators have expressed concern about the applicability of van den Berghe and colleagues' findings [4,5] to other settings [9–11]. These concerns include the relatively high mortality in relation to severity of illness among patients in the control group in one study [4]; the frequent administration of parenteral calories (which is unusual among most ICUs); the single-center design of the two studies [4,5]; and the fact that the investigators could hardly be blinded.

The results of two much larger trials are awaited (the GLUControl trial [12] and the NICE-SUGAR trial [13]). In the mean time, different experts give different recommendations: some argue that although the evidence for IIT does not yet support a grade-A recommendation (based on the highest level of evidence), it does appear to be stronger than the evidence in support of a strategy of tolerating hyperglycemia [10]. Another suggestion is just to target a BGC of over 150 mg/dl [9], or to reserve IIT solely for critically ill patients after elective surgery [11].

One of the most frequently mentioned and feared complications of IIT is hypoglycemia. Indeed, 5.1% of patients treated with IIT in surgical intensive care versus 0.7% of control patients developed severe hypoglycemia (BGC is defined as less than 40 mg/dl) [4]. In medical ICU patients, severe hypoglycemia occurred even more often with IIT: 18.7% of study patients versus 3.1% in the conventionally treated group encountered severe hypoglycemia [5]. Of note, the recent multicenter VISEP trial in Germany by the SepNet group was discontinued prematurely because of identical mortality rates in the treatment group and in the control groups but a higher incidence of hypoglycemia in the IIT group (12.1% versus 2.1%) [14]. Patients in the ICU who were sedated and patients with disturbances in the counter-regulatory responses to hypoglycemia are at risk for neuroglycopenia because of the absence of clinical symptoms of severe hypoglycemia. Neuroglycopenia may cause cerebral damage, epileptic insults, or even coma [15].

## Current Practice of IIT

### Surveys

McMullin and colleagues surveyed ICU nurses and ICU physicians on the blood-glucose-concentration thresholds that they acted upon in five university-affiliated multidisciplinary ICUs in Canada [16]. The reported clinically important threshold for hyperglycemia was remarkably high. Indeed, median threshold was 180 mg/dl (interquartile range [IQR] 162–216 mg/dl). The reported median clinically important threshold for hypoglycemia was 72 mg/dl (IQR 54–72 mg/dl). ICU nurses acted on slightly but significantly higher thresholds than ICU physicians (a difference of 9 mg/dl).

Avoidance of hyperglycemia was judged most important for patients with diabetes, a recent seizure, advanced liver disease, or acute myocardial infarction. Surprisingly, avoiding hyperglycemia was judged unimportant for surgical patients—the targeted patients in van den Berghe and colleagues' pivotal study on IIT in patients in the ICU [4]. In McMullin and colleagues' paper [16], the authors gave no information regarding presumed risks of IIT, in particular the risk for hypoglycemia and the impact of this risk on the chosen BGC thresholds.

Mackenzie and colleagues recently reported a survey on the use of IIT in large English hospitals [17]. Only 25% of ICUs reported blood-glucose-concentration targets to be similar to those used in the study by van den Bergh and colleagues [4]. Most ICUs in which IIT was performed reported higher normal blood-glucose-concentration limits. Interestingly, most ICU nurses (82%) reported being afraid of hypoglycemia in the patients receiving IIT [18].

Mackenzie and colleagues' findings are partly in line with a recent survey in the Netherlands by three of us (MJS, PES, and HSM) [19]. Over 100 participants of the annual meeting of the Dutch Society of Intensive Care were surveyed, most of them ICU physicians. Of the participants, 69% stated that IIT was already being applied in their ICU, while 7% mentioned they would start with this intervention shortly. Of those that said they applied IIT in their ICU, 62% used some sort of intensive insulin protocol with sliding scales. Twenty-six percent stated that their ICU used blood-glucose-concentration limits of 80–110 mg/dl, 73% stated that their ICU used limits of 80–145 mg/dl, and 2% stated that their ICU used limits of 80–180 mg/dl. Eighteen percent of respondents said that glycemic control was applied solely by ICU nurses, 16% said that it was applied by ICU physicians alone, and 65% said that it was applied by ICU nurses and ICU physicians as a team (1% did not answer the question).

Recently, the Australian and New Zealand Intensive Care Society Clinical Trials Group (ANZICS-CTG) conducted a practice survey [20]. There were 45 affiliated ICUs that were E-mailed a blood-glucose survey, enquiring as to their familiarity with the van den Berghe and colleagues' studies [4,5] and whether IIT had been adopted. If IIT had been adopted, respondents were asked to which groups of patients IIT was applied and the reasons for such selection. If IIT had not been adopted, respondents were asked their reasons for failure to adopt this strategy. Sixty-four percent of ICUs responded to this survey; all were familiar with the studies on IIT, but only 10.3% had adopted IIT in all their patients. In 31% of responding centers, IIT was applied in selected patient groups, predominantly those that stayed in the ICU for over three days, those with sepsis, and postsurgical patients. The reasons for not applying IIT were due to concerns about the risk of hypoglycemia and concerns about the external validity of the two studies by van den Berghe and colleagues.

Although these surveys may only be an incomplete reflection of practice throughout the world, the striking similarities between their results at least suggest that IIT is far from being part of the standard care of critically ill patients.

### Targets of intensive insulin therapy

Only two of the identified studies [21,22] used BGC targets identical to those used in the two studies by van den Berghe et al. [4,5] (Table 1). All other studies used different BGC thresholds, most of them with a higher upper limit (up to 150 mg/dl). Of interest, most studies found that higher BGC limits were deliberately chosen to facilitate acceptance of the protocol (i.e., because it was suspected that there would be an unacceptably high incidence of hypoglycemia when applying the limits used by van den Berghe and colleagues [4,5]).

**Table 1:**
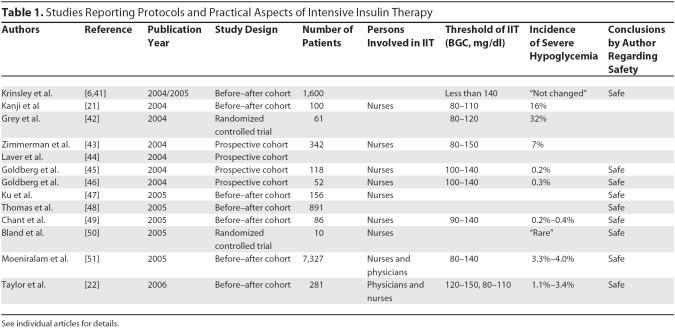
Studies Reporting Protocols and Practical Aspects of Intensive Insulin Therapy

### Incidence of hypoglycemia

The incidence of hypoglycemia varied from as low as 0.5% to as high as 18.7%, when using the threshold of 40 mg/dl (Table 1). When using BGC less than 60 mg/dl and less than 72 mg/dl as a threshold for hypoglycemia, incidences were 32% and 29%, respectively. However, since the reliability of capillary blood-glucose measurements (blood obtained from a finger stick) are unsatisfactory (there is a high degree of imprecision and a high percentage of discordance [23]) in many of the reviewed studies, the incidence of hypoglycemia may have been higher or lower than reported. In most reports, IIT was considered a safe strategy.

### Personnel involved in intensive insulin therapy

Although it was not always clearly stated in the papers, it seems that ICU nurses were the primary health-care workers involved in the application of the IIT protocol (Table 1). Only one study compared an ICU nurse-driven IIT protocol with a protocol applied by ICU physicians alone [22]. In this cohort study, three consecutive regimens were compared: IIT applied by ICU physicians with no specific targets, IIT applied by ICU nurses aiming at BGC between 120–150 mg/dl, and IIT applied by ICU nurses aiming at the BGC used by van den Berghe and colleagues [4,5]. There was a significant decrease in average daily BGC, from 190 to 163 to 131 mg/dl in the three consecutive phases of the study. The incidence of severe hypoglycemia (defined as blood glucose concentration less than 40 mg/dl) was similar between the groups, ranging between 1.1% and 3.4%. Remarkably, protocol compliance was reported to be low (only about 50% of orders were followed), and blood for BGC monitoring was at times obtained from a finger stick, which may be unreliable, as explained above.

## Discussion

A systemic approach to the implementation of research evidence in daily practice is recommended. Indeed, before an intervention is implemented, different phases of accumulating evidence with respect to the intervention should be followed. A framework for the implementation of research evidence that leads to understanding of barriers and opportunities involved in the implementation of protocols or guidelines in health care has been proposed [2,24].

Identifying barriers for IIT is an important part of the process of its implementation [1]. Fears and barriers should be catalogued and rationalized. In fact, implementation of complex strategies requires a thorough social investigation before such strategies will be applied in daily practice. Considering IIT, we now recognize several hampering factors: concerns about the external validity of the two studies by van den Berghe and colleagues [4,5], the potential increased risk of hypoglycemia with associated neurological damage, and uncertainties on how (and who is) to apply and monitor IIT.

### Factors hampering uptake of other ICU treatments

The hampering factors for the implementation of IIT in intensive care medicine are not unique, but are comparable to other strategies, both in intensive care medicine and in other medical specialties. This is nicely illustrated by the implementation processes of several strategies in the ICU in the last decade, such as the use of recombinant human-activated protein C (rh-APC) in severe sepsis [25], and the use of lung-protective mechanical ventilation in patients with acute lung injury [26] (Table 2).

**Table 2:**
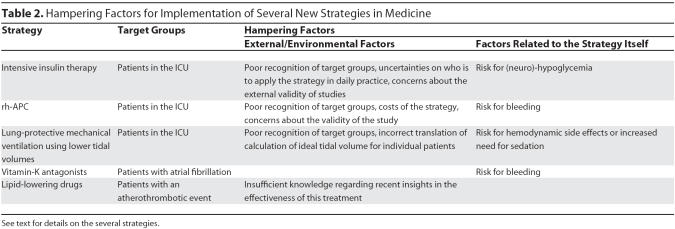
Hampering Factors for Implementation of Several New Strategies in Medicine

The use of rh-APC is hampered by two practical problems. First, the costs of APC treatment are high and are not well compensated at the hospital level, at least in several European countries [27]. Second, recognition of patients that may benefit from APC can be difficult, especially when a guideline has been developed aiming at restrictive use of APC. Another concern with the use of APC is the perceived increased risk of bleeding. It is difficult to understand how this perception arose, given the overall benefit of the treatment and the relatively low incidence of clinically significant major bleeding, such as intracerebral bleeding.

As regards the use of lower tidal volumes in patients with acute lung injury, one of the reasons why this strategy is not implemented in daily practice is simply that the calculation of tidal volumes is not adequately understood [28]. Also, patients with acute lung injury are often not easily recognized, causing many patients who may benefit from lower tidal volumes not to be ventilated in a lung-protective way [29]. Finally, many of the suggested side effects of the use of lower tidal volumes, such as the potential increase in use of sedatives or hemodynamic instability, are now recognized to be no more than imaginary [30,31].

### Factors hampering uptake of general medical treatments

In many other fields of medicine, implementation of evidence-based practice is difficult, as shown by the insufficient use of vitamin K–antagonist anticoagulant treatment in patients with atrial fibrillation [32,33] or the underuse of statins as secondary prophylaxis in patients with an atherothrombotic event [34,35]. In these cases, factors causing insufficient implementation may include (among others) fear of complications (such as bleeding in the case of anticoagulant therapy), insufficient knowledge regarding recent evidence on the effectiveness of these treatment options, or the additional workload for the physician, and the perceived burden for the patient caused by these interventions.

### IIT and hypoglycemia

The observed incidence of hypoglycemia in the reviewed studies varies greatly, and depends on the definition of hypoglycemia, the target range for BGC, and the way in which BGCs were monitored. However, reporting the incidence of a BGC below a particular figure may be counterproductive [18,19]. Information on the proportion of time spent in the target range, above the target range, and below the target range would be more useful when evaluating published reports on the efficacy and safety of IIT [36].

The most feared consequence of hypoglycemia is potentially irreversible neurological damage. How low does the hypoglycemia need to be, and for how long, for this complication to occur [18]? Repeated episodes of insulin-induced hypoglycemic coma for periods ranging from 45 minutes to three hours for treating opiate addiction and schizophrenia (in the 1940s) were found to have minimal long-term effects and a mortality of less than 1% [37]. In addition, long-term follow-up of patients with diabetes mellitus randomized in a large prospective trial of IIT failed to detect any association between the frequency of severe hypoglycemia and cognitive decline [38]. Only subtle, reversible impairments of attention could be detected in patients without diabetes undergoing dynamic pituitary function assessment using hypoglycemic stress with BGC of 29 mg/dl [39].

In two years of IIT, Mackenzie et al. recorded 128 instances of hypoglycemia (blood glucose concentration less than 40 mg/dl) out of 29,733 measurements, with a median value of 33 mg/dl (IQR 25–36 mg/dl) and a median duration of 18.2 minutes (8.4–37.5 minutes) [18]. The incidence of hypoglycemia in their study decreased significantly with time. The authors concluded that the risk of a patient suffering prolonged severe hypoglycemia was small and the risk of this resulting in significant neurological damage was even smaller.

In the two studies by van den Berghe et al., IIT in patients in the ICU was purely a nurse-driven protocol [4,5], but it is questionable whether nurses really want to adopt IIT, especially when the targets are set at the lower normal limits of BGC. In these studies, the nurses were dedicated research nurses, who may differ in their attitude toward IIT compared with routine ICU nurses. From personal experience, we know that nurses now and then might abandon the protocol, in particular at the lower normal limits, with the intention of preventing hypoglycemia.

One survey suggested that IIT might be better if applied by ICU physicians [19]. However, in the one study that compared ICU physicians with ICU nurses [22], no differences in respect to safety (incidence of hypoglycemia) and efficacy (average daily blood glucose concentration) were seen. In addition, ICU nurses' continuous presence at the bedside may prevent deterioration of glucose control. Indeed, many of the predisposing factors for hypoglycemia in patients in the ICU are easily recognizable by ICU nurses, such as worsening nutritional status without adjustment for insulin infusion [40]. Future studies should focus on how to implement IIT in daily practice, putting special emphasis on which health professionals in the team should actually apply IIT guidelines, and to what extent.

## Conclusion

At present, IIT is far from being standard practice. In addition, in those centers that report on their experiences with IIT, thresholds are set higher than in the two studies by van den Berghe and colleagues [4,5]. Several factors hamper the implementation of IIT, which are similar to factors that hinder the implementation of other evidence-based strategies. Nevertheless, it is promising to see the large number of studies reporting on implementation of nurse-driven IIT in critically ill patients. In addition, several larger studies on the efficacy of IIT are underway. When the process of critical appraisal of the published and upcoming trials on of IIT has finished, the time will have come to critically appraise implementation of IIT in daily practice.

## Supporting Information

Text S1 Search Strategy (21 KB DOC).

## Abbreviations

BGC: blood glucose concentrations
ICU: intensive care unit
IIT: intensive insulin therapy
MeSH: medical subject heading
rh-APC: recombinant human-activated protein C
