# Supplementary material for: Intensive Insulin Therapy in Intensive Care: An Example of the Struggle to Implement Evidence-Based Medicine
Source: PLoS Med. 2006 Dec 5;3(12):e456. doi: 10.1371/journal.pmed.0030456 (PMC1762062; doi:10.1371/journal.pmed.0030456)
Supplement: Text S1 — (21 KB DOC). [file pmed.0030456.sd001.doc]

**Text S1.** Search Strategy

The Medline database was used to identify medical subject headings (MeSH) to select search terms. In addition to the MeSH terms, we also used free-text words. Search terms referred to aspects of the population (“critical care” [MeSH], “intensive care”) and the condition (“insulin” [MeSH] OR “intensive insulin therapy” OR “intensive glucose control” OR “tight glycemic control” OR “strict blood glucose control” OR “strict glucose control” OR “insulin therapy”). These were combined with the terms “nurse-driven” and “physician-driven” and “clinical protocols” (MeSH) OR “guidelines” (MeSH) OR “nomograms” (MeSH). The relevance of each paper was assessed using the abstracts. Reports on so-called closed-loop computer systems were ignored, as were those solely reporting on computer models for IIT. Studies solely dealing with patients with diabetes mellitus were not taken into consideration. Also, the papers by van den Berghe were excluded [1,2]. The combination of the searches resulted in the identification of 40 manuscripts of potential interest. From the search, we identified four papers on current practice of IIT [16,17,19,20]. These manuscripts reported on recently performed national surveys in Canada, England, the Netherlands, and Australia/New Zealand, respectively. The search further identified 14 potentially relevant articles on IIT protocols [6,21,22,41–51]; two of these latter reports turned out to be on one single study [6,41]. In total, 13 studies with data from more than 13,000 patients were further analyzed. Seven of the analyzed studies were combined retrospective–prospective cohort studies (i.e., with a historical control group) [21,22,41,47–49,51], four were prospective cohort studies [43–46], and two were randomized controlled trials [42,50]. Of the 11 nonrandomized studies, nine studies compared glucose control before and after the implementation of some sort of IIT protocol [21,22,41,43,44,47–49,51], and two studies simply evaluated the implementation of IIT (i.e., a control group was lacking) [45,46]. Only one study directly compared a nurse-driven IIT strategy with a physician-driven strategy [22].
